# SHYCD induces APE1/Ref-1 subcellular localization to regulate the p53-apoptosis signaling pathway in the prevention and treatment of acute on chronic liver failure

**DOI:** 10.18632/oncotarget.19891

**Published:** 2017-08-04

**Authors:** Jianxin Diao, Haiye Li, Wei Huang, Wenxiao Ma, Huan Dai, Yawei Liu, Ming Wang, He Yu Hua, Jinying Ou, Xiaomin Sun, Xuegang Sun, Yungao Yang

**Affiliations:** ^1^ School of Traditional Chinese Medicine, Southern Medical University, Guangzhou, China; ^2^ Gao Ming People’s Hospital, Foshan, Guangdong, China; ^3^ Nanfang Hospital, Southern Medical University, Guangdong,Guangzhou, China; ^4^ Zhujiang Hospital of Southern Medical University, Guangdong, Guangzhou, China

**Keywords:** San huang yin chi decoction, acute on chronic liver failure, APE1/Ref-1, apoptosis, p53, Pathology Section

## Abstract

*Background & Aims: San huang yin chi* decoction(SHYCD) is derived from the yin chen hao decoction, a well-known and canonical Chinese medicine formula from the “Treatise on Febrile Diseases”. Over the past decade, SHYCD has been used to treat and prevent the liver cirrhosis and liver failure. In the present study, we investigated the effects of SHYCD for acute on chronic liver failure(ACLF) and explored its potential mechanism. an ACLF rat model, which induced by carbon tetrachloride (CCl4) combined with D-galactosamine (D-GalN) and lipopolysaccharide(LPS), was used and confirmed by B-ultrasound analysis. Rats were randomly divided into control group, model group, SHYCD-H group, SHYCD-M group, SHYCD-L group, AGNHW group. Compared with the ACLF model group, High, medium, and low doses of SHYCD reduced ALT, AST, TBIL, NH3, IL-1β, IL-6, and TNFα expression levels in the serum, Shorten PT and INR time,and increased Fbg content in the whole blood, increased survival rate of the rats, improved liver pathological changes. APE1 / Ref-1 was mainly expressed in the nucleus, but the nucleus and cytoplasm were co-expressed after hepatocyte injury. SHYCD significantly downregulated APE1/Ref-1 expression in the cytoplasm. Increased APE1/Ref-1, Bcl-2, reduced p53, caspase-3, Bax, and Cyt-c in the total protein. Base on the results, we conclused that High, medium, and low doses of SHYCD could be applied in prevention and treatment of ACLF, and dose-dependent. The possible mechanism is to promote the APE1 / Ref-1 from the cytoplasm to the nuclear transfer, regulation of p53 apoptosis signal pathway prevention and treatment of ACLF.

## INTRODUCTION

The pathogenesis of acute on chronic liver failure(ACLF) is complicated and the mortality is high [1].In recent years, many domestic studies have shown that the death rate of drug-induced hepatic failure has increased to 63.82% [2]. A study by the Royal College of Medicine showed that the mortality of ACLF is approximately 60% to 70% [3]. Currently, Western medicine still lacks specific drugs and treatment methods for ACLF. The ultimate therapy for cirrhosis and end-stage liver disease is liver transplantation [4]. However, the Chinese medicine treatment for hepatitis B-related ACLF has a significant curative effect [5]. The Chinese herbal medicine, *QingganHuoxue,* significantly improved liver function and increased survival rates in rats with acute liver failure [6]. The *Sanhuangyinchi* decoction (SHYCD) and *Angongniuhuang* pill(AGNHW) improved liver function and inhibited caspase-3 activity in rats [7].Point application with Angong Niuhuang sticker protects hippocampal and cortical neurons in rats with cerebral ischemia [8]. Traditional Chinese medicine has certain curatives effect on the prevention and treatment of severe hepatitis and ACLF.

SHYCD is composed of Artemisia *capillaris*, rhubarb, turmeric, Astragalus *membranaceus*, and *Radix Paeoniae rubra*. SHYCD has fever-reducing and detoxifying effects. SHYCD also promotes blood circulation to remove blood clots and increases immunity [7]. The main bioactive compounds in Artemisia capillaris are volatile oil, artemisia, coumarin, flavonoids, chromogen ketones, and chlorogenic acid. These compounds protect liver cell membranes, prevent liver cells necrosis, promote liver cell regeneration, and improve liver microcirculation [9]. The main bioactive compound in rhubarb is an anthraquinone derivative, which is used in the treatment of severe hepatitis [10]. The main bioactive compound in Radix Paeoniae Rubra is paeoniflorin, which induces LoVo cell apoptosis by increasing intracellular calcium concentrations [11]. The main bioactive compounds in Astragalus are saponins, astragalus polysaccharides, and flavonoids. Flavonoids have antioxidant activities. The main [12] bioactive compounds in turmeric are curcumin and volatile oils, which have anti-oxidant, anti-inflammatory, anti-cancer, and free [13, 14]-radical scavenging activities. Our preliminary study demonstrated that SHYCD reduced AST, ALT, and TBIL levels and cleaved caspase-3 protein expression [15]. However, the mechanism of action of SHYCD is still unclear and needs further study. Apurinic/apyrimidinic endonuclease/redox effector factor-1 (Ape1/Ref-1) is a protein with multifunctional roles in cells impacting a wide variety of important cellular functions [16]. Ape1/Ref-1 forms a unique link between the DNA BER pathway,cancer, transcription factor regulation, oxidative signaling, and cell-cycle control [16, 17]. Recent developments have also implicated Ape1/Ref-1 as a major controlling factor in p53 activity through redox -dependent and redox-independent mechanisms [18]. Ape1/Ref-1 is closely linked with apoptosis [19] and altered levels or altered cellular localization of Ape1/Ref-1 have been found in some cancers, including ovarian, cervical, prostate, and germ-line cell tumors [20]. New research shows that the pathophysiology of ACLF is related to persistent inflammation, immune dysregulation with initial widespread immune activation, and cell apoptosis [21]. Cytokines are environment important molecules that inhibit the extracellular of hepatocytes. TNF-α, IFN-γ, IL-1 and IL-6 are involved in the pathogenesis of ACLF [22]. We hypothesize that APE/Ref-1 may play a key role in ACLF by inhibiting the pro-apoptotic protein p53, thereby reducing liver cell apoptosis by regulating cell apoptosis-related proteins. Based on the above assumptions, the present study will investigate the mechanism of SHYCD in apoptosis.

## RESULTS

### Effects of SHYCD on liver, spleen, ascites and survival rate

**Figure 1:**
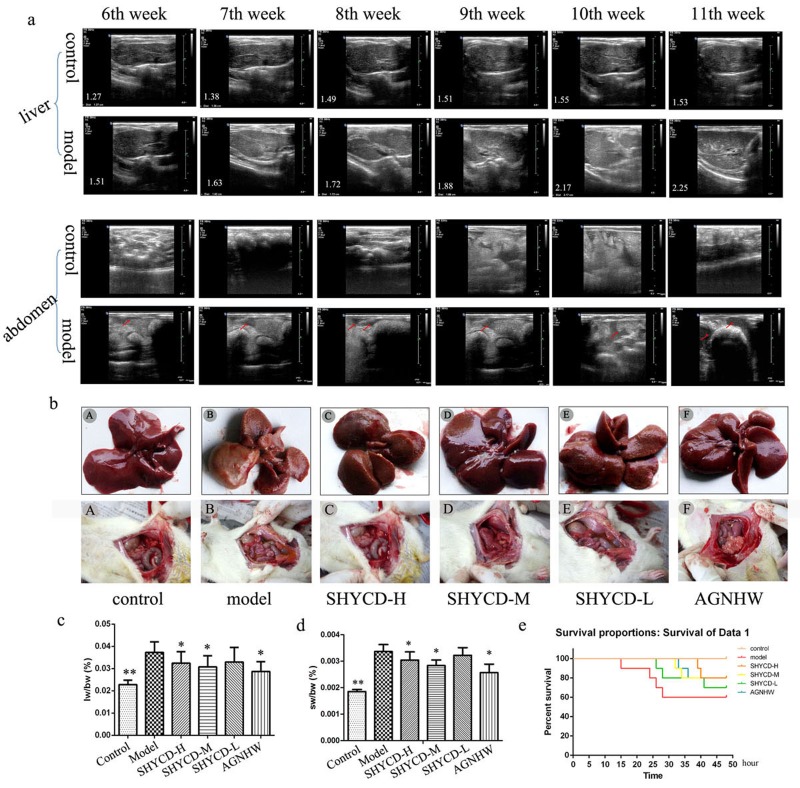
Modeling effects in rats and SHYCD effects on rat survival state **a.** B -Ultrasound imaging results for rat liver, spleen size, and number of abdominal ascites. **b.** Macroscopic observation of the liver gloss, color, form, and number of abdominal ascites. **c.**, **d.** Comparison between the ACLF model group and control group showing that liver and spleen weight ratio to body weight increased significantly in ACLF model group (**P < 0.01; *P < 0.05, vs. model). **e.** Observation of ACLF rat model group survival curves within 48 hours based on carbon tetrachloride toxicity and joint D -GalN and LPS acute attack mimicking ACLF.

The individual rats exhibited hepatomegaly and a few ascites during the first 6 weeks. Hepatomegaly and ascites became more obvious over time. B-ultrasound screening showed 50 positive rats out of the 60 rats injected with 40% CCl_4_ peanut oil solution for a screening rate of 83.3% (Figure 1a). Livers from the control group were smooth and bright with complete lobular architecture. Livers from the ACLF model group were enlarged with obvious ascites, lobular structural damage, and nodular liver morphology. Livers from groups treated high, medium, or low doses of SHYCD groups had improved liver morphology and reduced ascites (Figure 1b). Compared with the control group, the liver and spleen weight to body weight ratios of the treated groups increased significantly (**P<0.01; *P<0.05) (Figure 1c, 1d). The Kaplan-Meier survival curves showed that SHYCD treatment significantly increased survival rate of the rats (Figure 1e).

### Effects of SHYCD on ALT, AST, and TBIL serum levels and on PT, INR, Fbg, and NH3 whole blood levels

**Figure 2:**
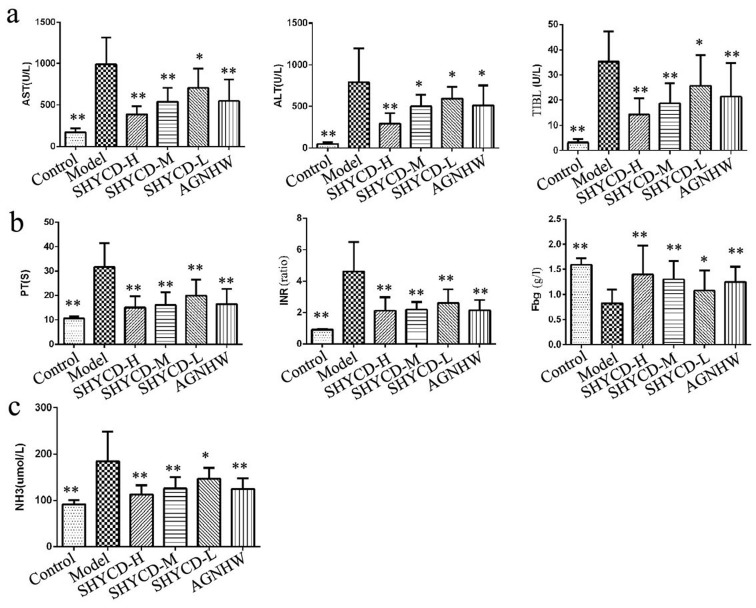
The influence of SHYCD on rat liver function, blood coagulation, and blood ammonia levels **a.** Values for ALT, AST, and TBIL as the three indicators of liver function. **b.** Values for PT, INR, and Fbg as the three indicators of coagulant function. **c.** Blood ammonia level. *p < 0.05; **p < 0.01 VS model.

ALT, AST, TBIL, Fbg, and NH3 significantly increased, whereas PT and INR time were significantly prolonged in the ACLF model group (*P* < 0.05). All these parameters indicate severe liver damage. SHYCD high, medium, and low doses reduced the serum expression levels of ALT, AST, TBIL, and NH3, shortened PT and INR time, and increased Fbg content (*P* < 0.01). These effects were most obvious in the groups treated with high or medium doses of SHYCD. The positive control drug Angongniuhuang also had considerable effect. SHYCD prevented ACLF in a dosage-dependent manner. (Figure 2)

### Effects of SHYCD on IFN-α, IL-1, IL-6, and TNFα serum levels

Serum IFN-α, IL-1β, IL-6, and TNF-α expression were upregulated in the ACLF model group and were significantly inhibited by SHYCD. The high dose group exhibited the most obvious effect (*P<0.01) (Table1).

**Table 1:** Effects of SHYCD surem cytokines IFN-γ, IL-1β, IL-6, TNFa (x±s).

Group	n	IFN-γ (pg/L)	IL-1β (pg/L)	IL-6 (pg/L)	TNFa (pg/L)
Control	10	34.33±19.6*	26±5.3*	13.2±7.6*	15.0±1.0*
Model	6	1365±110.1	683±41.9	209.7±121.0	1835.3±266.8
SHYCD-H	8	219.8±94.0*	231.8±19.8*	118.2±68.2*	664.0±160.8*
SHYCD-M	8	650.2±112.2*	336.8±44.7*	82.4±47.6*	800.0±76.4*
SHYCD-L	7	756.7±204.2*	383.6±37.0*	40.4±23.3*	1117.3±97.5*
AGNHW	8	593.3±144.8*	311±24.3*	47.1±27.2*	818.7±101.0*

*P<0.01, *vs* model.

### Effects of SHYCD on the histological and ultrastructural changes in the liver

**Figure 3:**
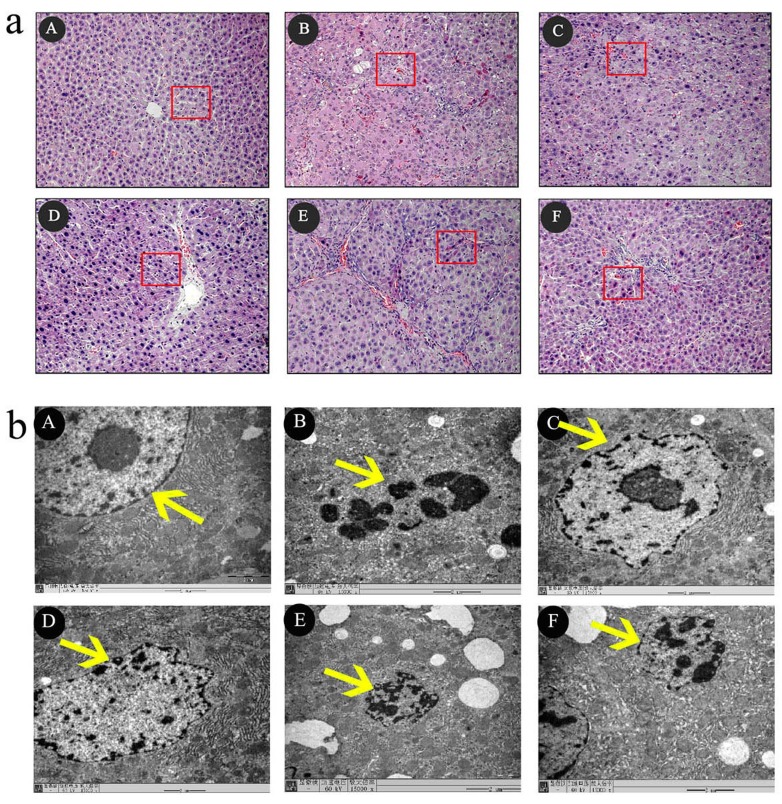
The effects of SHYCD for ACLF on the Histological and Ultrastructural changes in the liver tissue **a.** Representative microscopic images of HE stain. 200x **b.** TEM images of ultrathin sections of myocardial tissue are changed,15000x. A: control group;B:Model group; C:SHYCD-H group; D: SHYCD-M group; E: SHYCD-L group; F:AGNHW group.

Light microscopy showed that liver sections from the control group had clear lobular

structures and that liver cells were arranged in an orderly manner. Liver sections from the ACLF model group showed liver cell necrosis, disordered liver cell arrangement, lobular fusion structure, obvious hepatic sinus expansion and bleeding, lobules, periportal inflammatory cell infiltration, and liver cell edema. Treatment with high, medium, or low doses of SHYCD attenuated liver pathological damage (Figure 3a). Electron microscopy (SEM) images of liver sections from the ACLF model group showed the disappearance of cell membrane amalgamation, visible apoptotic bodies, mitochondrial swelling, membrane damage, fractured or missing crest, and obvious nuclear pycnosis. Treatment with high, medium, or low SHYCD doses improved the ultrastructure of liver cell. SEM images from the SHYCD-treated groups showed reduced or no apoptotic bodies, subsided mitochondrial swelling, increased crest, and relatively complete nuclear structures (Figure 3b).

### Effects of SHYCD on APE1 mRNA and protein levels

**Figure 4:**
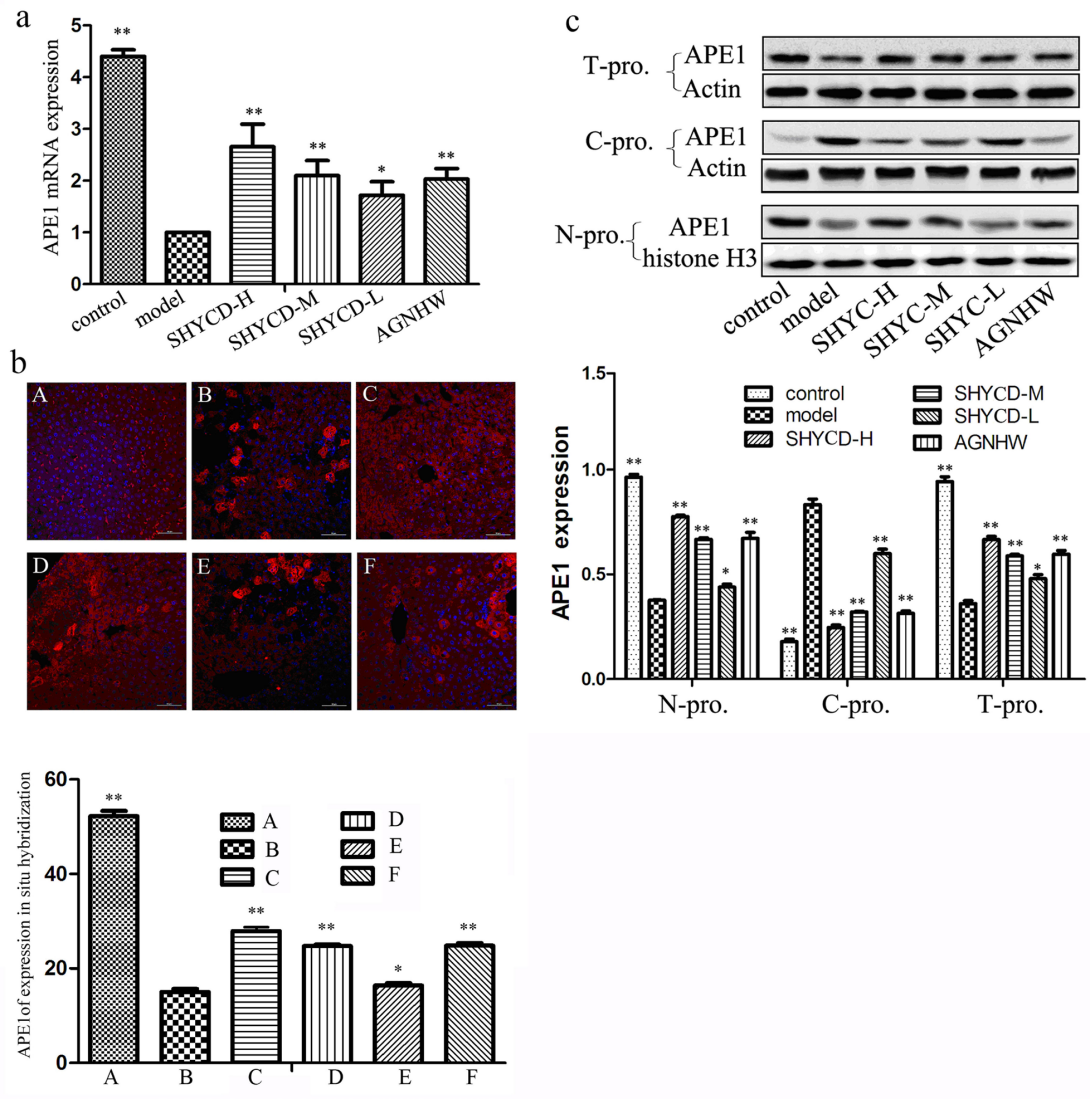
The effects of SHYCD on APE1 mRNA and protein levels **a.** Effect of SHYCD on APE1 mRNA **b.** Confocal immunofluorescence microscopy image of APE1 in situ hybridization (400x). A: control group; B: model group; C:SHYCD-H group; D: SHYCD-M group; E: SHYCD-L group; F:AGNHW group,*p < 0.05; **p < 0.01,VS model. **c.** Western blot of APE expression in liver tissue nucleoprotein and cytoplasm protein. N-pro: nucleoprotein, C-pro: cytoplasm protein, T-pro:total protein. *p < 0.05; **p < 0.01,VS model.

Quantitative PCR results showed that APE1/Ref-1 mRNA levels decreased in the ACLF model group. However, groups treated with high, medium, or low SHYCD doses showed upregulated APE1/Ref-1 mRNA expression levels (*P*<0.01) compared with the control group. Confocal immunofluorescence microscopy images of in situ hybridization showed that in the control group, APE1/Ref-1 expression occurred mainly in the nuclei of liver cells (Figure 4a). In the ACLF model group, APE/Ref -1 expression was localized in the nucleus and co-expressed in the plasma. Necrotic cells did not express APE/Ref-1. Compared with the control group, APE1/Ref 1 expression in the cell plasma of the ACLF model group increased significantly, whereas APE1/Ref-1 expression in the nucleus decreased significantly. Treatment with high, medium, or low doses of SHYCD reduced the cytoplasmic expression of APE1/Ref-1 and increased the nuclear expression of APE1/Ref-1 (Figure 4b). Western blot further confirmed that APE1/Ref-1 was mainly expressed in liver cell nuclei, and that APE/Ref-1 expression in the model group was localized in the nucleus and was co-expressed in the plasma. These results were consistent with the immunofluorescence results (Figure 4c).

### APE1 interaction with p53

**Figure 5:**
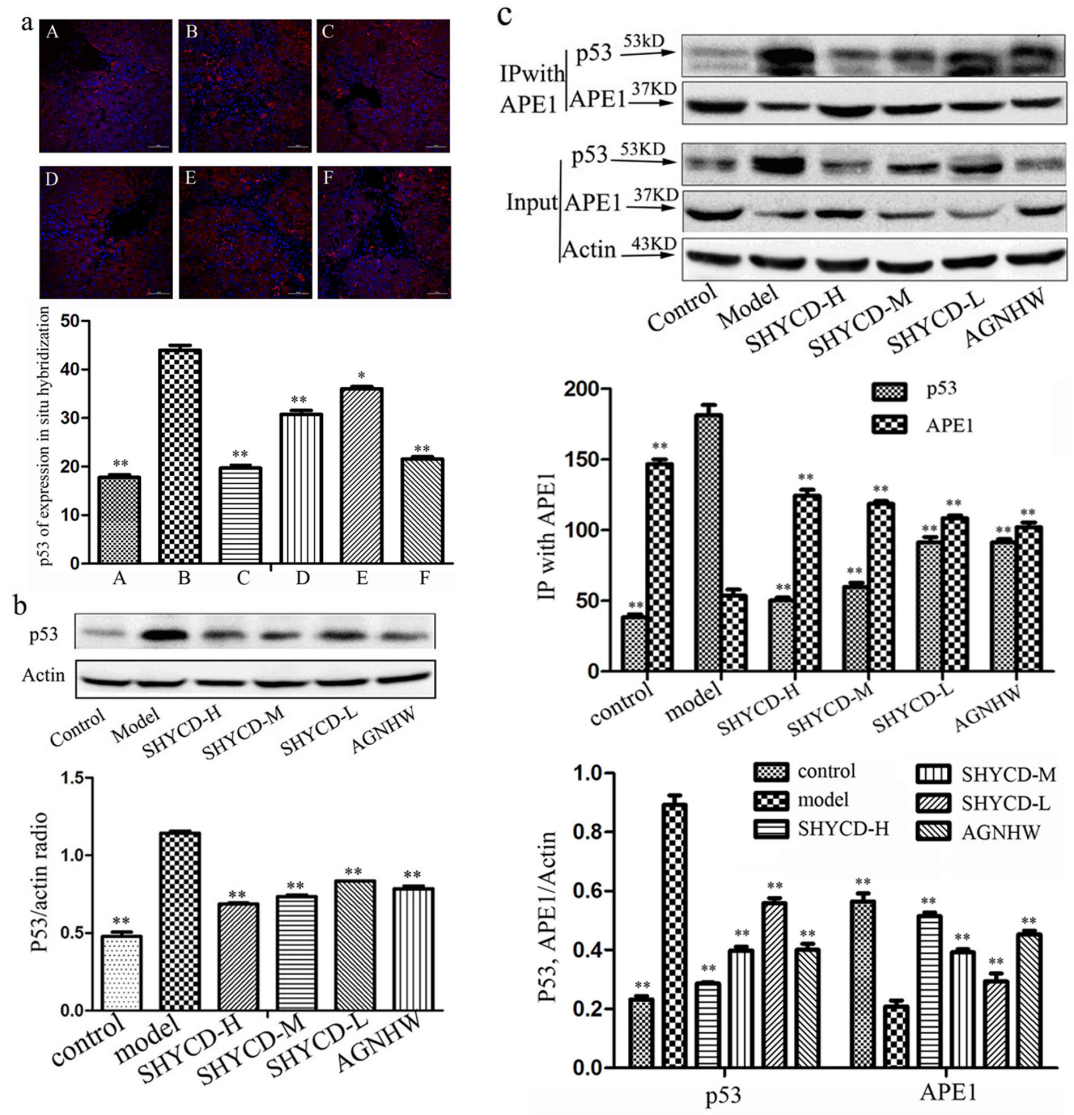
APE1 interaction with P53 **a.** Confocal immunofluorescence microscopy image of p53 in situ hybridization (400x). A: control group; B: model group; C:SHYCD-H group; D: SHYCD-M group; E: SHYCD-L group; F:AGNHW group,*p < 0.05; **p < 0.01,VS model. **b.** Western blot analysis of p53 expression,*p < 0.05; **p < 0.01,VS model. (c) IP performed with an anti-APE1 antibody. Co-IP P53 and APE1 were detected by Western blot.

Confocal immunofluorescence microscopy images of in situ hybridization showed lower p53 expression in the control group, while expression levels were higher in the ACLF model group. Treatment with high, medium, or low doses of SHYCD reduced p53 protein expression. Reduced p53 protein expression was the most obvious in the high-dose group. The groups treated with a mediumdose of SHYCD and the positive control drug Angongniuhuang also showed considerable effects (*P* < 0.01) (Figure 5a). Western blot results also showed that p53 protein expression was lower in the control group and higher expression of p53 in the model group. The effects of each SHYCD dose level were consistent with the results of immunofluorescence in situ hybridization (Figure 5b). Immune coprecipitation further confirmed the interaction between p53 and APE1 protein, indicating that p53 might be regulated by the multifunctional APE1 protein (Figure 5c).

### SHYCD reduced Bax, cleaved caspase-3, and Cyt-C protein expression, increased BCL-2 protein expression, and inhibited hepatocyte apoptosis

**Figure 6:**
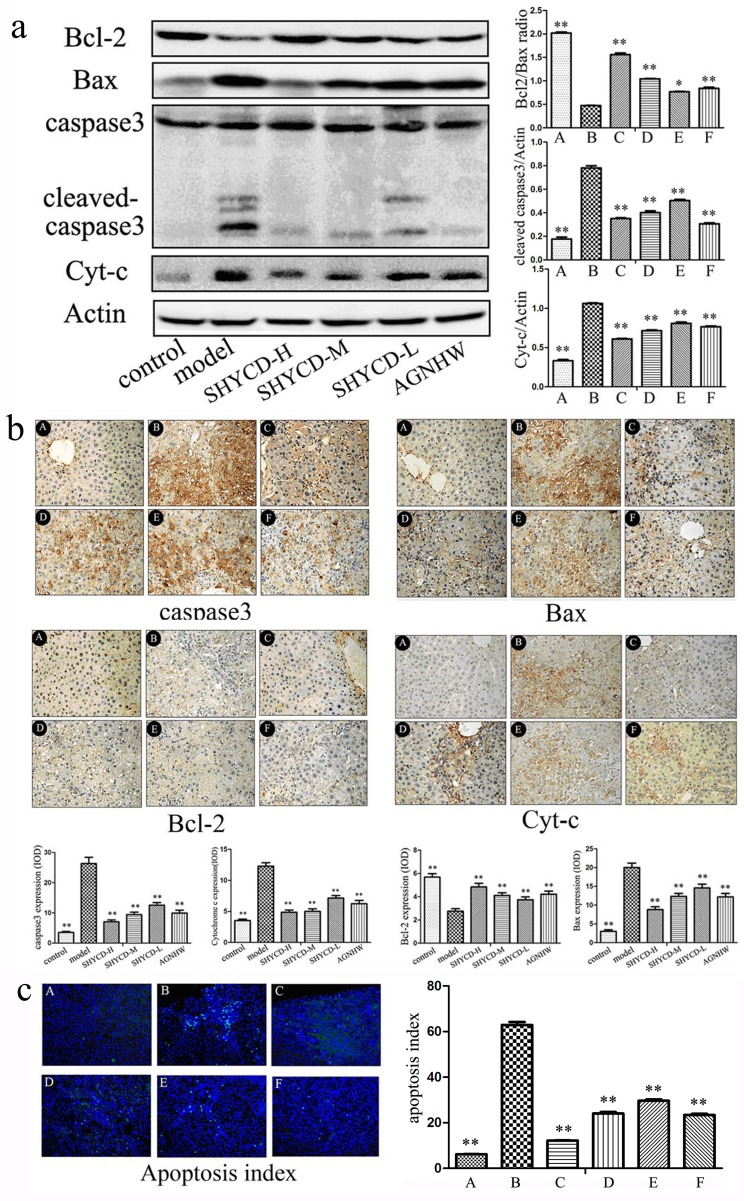
SHYCD reduced Bax, cleaved caspase-3, and Cyt-C, and increased BCL-2 SHYCD also inhibited hepatocyte apoptosis **a.** Western blot results showing that SHYCD reduced Bax, cleaved caspase-3, and Cyt-C, and increased BCL-2. **b.** Immunohistochemistry results showing that SHYCD reduced Bax, cleaved caspase-3, and Cyt-C, and increased BCL-2 (200x). **c.** Tunel results showing that SHYCD reduced hepatocyte apoptosis (200x). *p < 0.05; **p < 0.01, VS model. A: control group; B: model group; C:SHYCD-H group; D: SHYCD-M group; E: SHYCD-L group; F:AGNHW group.

Western blot results showed that Bax, cleaved caspase-3, and Cyt-C protein expression increased and BCL-2 protein expression decreased in the ACLF model group. The ratio of Bax and Bcl2 significantly decreased compared in the ACLF model group compared with the control group. Treatment with high, medium or low doses of SHYCD reduced Bax, cleaved caspase-3, and Cyt-C expression, increased BCL-2 expression, and improved the ratio of Bax and Bcl (Figure 6a). Immunofluorescence in situ hybridization showed that Bax, caspase -3, and Cyt-C significantly increased, whereas Bcl-2 significantly decreased, in the ACLF model group. These results were consistent with the Western blot results (Figure 6b). Tunel detection results showed that apoptosis rarely occurred in the control group, whereas apoptosis occurred significantly in the ACLF model group. Compared with ACLF model group, treatment with high, medium, or low doses of SHYCD reduced liver cell apoptosis (Figure 6c).

## DISCUSSION

Acute on chronic liver failure(ACLF) in chronic liver disease associated with acute or subacute hepatic decompensation clinical syndromes occur in the short term. The histopathology of livers from the ACLF model group showed liver necrosis, lobular structural damage, and dissolved nuclei, indicating extensive inflammatory infiltration and hepatocyte apoptosis [25, 26]. The joint treatment by carbon tetrachloride (CCl4) combined with rats in the ACLF model group had enlarged livers and obvious ascites, indicating chronic liver disease

D-galactosamine(D-GalN) and lipopolysaccharide(LPS) successfully established the ACLF model. CCl4 leads to the loss of function of liver cell enzymes and lipid peroxidation, (Figure 1). D-GalN, an amino sugar, is a hepatotoxic drug and inhibits uridinecausing liver cell damage and necrosis [27]. B -ultrasound imaging showed that after 11 weeks, metabolism, blocking RNA synthesis and leading to acute liver cell injury[28]. LPS induces neutrophil activation and accumulation, thus aggravating liver inflammation. Neutrophils also act on sinusoidal endothelial cells and capillaries. Neutrophils activate the endogenous coagulation system, resulting in liver microcirculation, which then leads to ischemia and hypoxia-induced liver injury [29]. Depressed RNA synthesis and the activation of inflammatory cytokines result in microcirculation -induced acute liver injury [30]. Figure 1 shows the enlarged livers, obvious ascites, lobular structural damage, and nodular liver morphology of the ACLF rat model group. Table 1 shows that serum alpha interferon(IFN-α), interleukin-1 beta (IL-1β), interleukin-1 (IL-6), and tumor necrosis factor-α(TNF-α) were upregulated in the serum of the ACLF model group. CCl4 combined with D-GalN and LPS established an ACLF model similar to the clinical onset of the disease process. Similarity to the clinical disease process of the animal model necessary to monitor the pathogenesis of the disease.

Liver injury is a precondition of liver failure. Clinical evaluation indices of liver failure include aminotransferase (AST), alanine aminotransferase(ALT), and total bilirubin(TIBL) levels. Liver function indicators are checked during routine health check- [31]ups. Figure 2a shows that ALT, AST, and TBIL levels significantly increased in the ACLF model group, whereas groups treated with high, medium, or low doses of SHYCD had reduced ALT, AST, and TBIL levels. This result indicates that SHYCD could enhance liver function. Coagulation disorders are an important cause of liver failure by causing liver microcirculation, such as hepatic decompensation [32]. Clinical evaluation indices for coagulation are prothrombin time (PT), international normalized ratio (INR), and Fbg. PT, as an exogenous coagulation system, is the more sensitive and most commonly used screening index. PT is a very important prognostic indicator of liver damage. PT not only indicates normal, extrinsic coagulation, but it also reflects liver synthetic function and reserve and the severity of liver damage [33]. Figure 2b shows that treatment with high, medium, or low doses of SHYCD shortened PT and INR time and increased Fbg content. Liver synthesis of urea decreases after liver failure, resulting in the increased intestinal release of ammonia directly into the systemic circulation and resulting in increased blood ammonia (NH3) [34]; thus, ammonia is an important index of liver function. Figure 2c results show that NH3 significantly increased in the model group, indicating severe liver damage. However, treatment with high, medium, or low doses of SHYCD reduced NH3 serum levels. Liver function, coagulation function, and blood ammonia results showed that treatment with SHYCD improved liver function, promoted blood clotting, and improved liver microcirculation

The body produces endotoxins to activate TNFα release during liver failure. TNFα stimulates related cytokines and the cytokine cascade reaction. TNFα is the most important inflammatory cytokine in liver cell injury, with a wide range of biological effects. TNFα stimulates IL-1, IL-6, and other cytokines, the release of reactive oxygen species, and a variety of other enzymes. TNFα promotes the aggregation of granulocytes and mediates inflammatory response after liver cell damage and multiple organ tissue injury [35]. TNFα, along with FasL, also binds to intracellular death receptors. TNF and Fas receptor-associated death domain proteins subsequently activate initiator caspase8. Caspase8 induces apoptosis through the direct activation of effector caspases3, 6, and 7 [36]. The combination of IL-1β and TNFα increases cell sensitivity to the effects of TNFα, increasing TNFα-induced tissue and cell damage and promoting the synthesis of acute phase proteins in the liver [37]. IL-6 is closely related to the pathological and physiological processes of many diseases, and promotes neutrophil activation and aggregation. Serum IL-6 levels directly reflect the extent of tissue damage.The Ape1/Ref-1 down-regulating expression could influence inflammation and the release of the inflammatory cytokines [22]. Our results showed that liver inflammation in rats was improved by SHYCD, as evidenced by decreased blood and liver levels of IL-1 β, IL-6, and TNF-α. These results suggested that SHYCD could improve liver failure via the inhibition of mitochondrial ROS, apoptosis, and anti-inflammatory effects

Apoptosis is a prominent feature of liver disease. Mechanisms of hepatic apoptosis are complicated by multiple signaling pathways [38]. The apoptotic process comprises the startup period, the effector period, and the washout period. Each period has a corresponding molecular mechanism. During the startup period, the main factors in the following categories initiate apoptosis: TNFR family death receptor activation, DNA damage stress signal generation, and signal generation survival. DNA damage causes p53 activation, and Bcl-2 family proteins regulate the apoptosis signal. The effector period involves the central part of the activation of apoptosis and mitochondrial permeability transition. Death substrate hydrolysis and chromosome fragmentation of phagocyte apoptotic bodies [39] occur during the wash-out period. These three processes eventually leads to apoptosis.

Although the relationship of Ape1/Ref-1 to apoptosis has been studied extensively, our study is the first to report that the altered expression of Ape1/Ref-1 is involved in ACLF. Immune fluorescence in situ hybridization results showed that Ape1/Ref-1 expression significantly increased in the cytoplasmic protein of the ACLF model group; however, the mechanism mediating this response is not fully understood. Further observations showed that Ape1/Ref-1 was expressed in the cytoplasm of damaged cells, but not in cytoplasm of necrotic cells. We performed nuclear plasma protein separation and Western blotting to validate these results. Nuclear plasma protein separation and Western blotting showed that cytoplasmic proteins in the ACLF model group increased Ape1/Ref-1 expression but not in the control group. Compared with the control group, nucleoprotein Ape1/Ref-1 expression was reduced. Ape1/Ref-1 is a multifunctional protein that is responsible for repair of DNA injury and also functions as a reduction-oxidation (redox) factor maintaining transcription factors in an active reduced state [40]. Ape1/Ref-1 regulates apoptosis, signal transduction, and the redox transcription factorsAT-1, P53, HIF-1, STAT3, NF-kB, and Mvb[41]. Ape1/Ref-1 is also involved in liver cell DNA base excision repair and in the regulation of apoptosis, resulting in increased expression in the plasma and decreased expression in the nucleus. PCR results and WB detection of total protein showed that the ACLF model group decreased APE1 /Ref-1 gene and protein expression compared with control group. APE1/Ref-1 is the limiting factor of the enzymatic DNA base excision repair pathway involved in DNA damage repair [42]. Despite the increased Ape1/Ref-1 expression in the cytoplasm, the reduced Ape1/Ref-1 expression was more obvious in the nucleus, resulting in the reduced total protein expression of Ape1/Ref-1 (p<0.05). In the ACLF model group, APE1/Ref-1 expression in the cytoplasm increased and may be involved in the regulation of mitochondrial function, activation of pro-apoptotic protein p53, and intervention in the apoptotic pathway. Figure 4 shows that treatment with high, medium or low doses of SHYCD increased APE1/Ref-1 expression in total protein and nucleoprotein, and reduced APE1/Ref-1 expression in the cytoplasm. Ape1/Ref-1 is a multifunctional protein that is not only responsible for repair of DNA damage, but also the regulation of apoptosis in ACLF.

Normal p53 gene or wild p53 gene (wt-p53) could promote apoptosis. p53 protein causes cell cycle arrest, induces apoptosis, and promotes the differentiation of biological functions [43]. P53 protein is the major receptor of cellular damage and cell apoptosis signal transmissions and could determine the state of DNA in the cell. If DNA damage is detected, p53 gene expression is increased and cell proliferation is terminated to prioritize DNA damage repair. However, if DNA is severely damaged or could not be repaired, p53 protein levels will still continue to increase, resulting in cell apoptosis [44]. Figure 5a and Figure 5b show that the control group had lower p53 expression, whereas the ACLF model group had higher p53 expression. Treatment with high, medium, or and low doses of SHYCD reduced p53 protein expression. The cytoplasmic expression of p53 and APE1/Ref-1 were similar. Both p53 and APE/Ref1 promote apoptosis. P53 detects the state of DNA in cells, while APE1/Ref -1 repairs DNA damage, indicating that DNA repair and apoptosis regulation are closely linked. Immune coprecipitation further confirmed the interaction between p53 and APE1/Ref -1. APE1/Ref-1 may inhibit pro-apoptotic protein p53 and regulate apoptosis pathway proteins.

P53 regulates caspase-3, Bcl 2, and Cyc-t. Additionally, p53 induces apoptosis Bax and cyclin G [45] gene activation. Because Bax is the target of p53 transcription, p53 can induce apoptosis by increasing Bax transcription. The Bcl-2 family of proteins simultaneously inhibits apoptosis and protects p53-mediated apoptosis [46]. The Bcl-2 family of proteins is associated with caspase activation factors and dimer formation. Activated caspase precursors are synthesized on the mitochondrial membrane pores and act to control the release of the activators in the mitochondria, regulate cell survival, and apoptosis [47]. Mitochondrial damage occurs when cellular antioxidants are depleted and ROS accumulation reaches a critical threshold. This process leads to the release of cytochrome c (Cyt-c) from the mitochondria, which then activates the caspase apoptotic pathway[30]. Figure 6 shows that in the ACLF model group, Bax and Cyt-c protein expression increased, whereas Bcl-2 protein expression decreased. The ratio of Bax and Bcl2 also significantly decreased compared with the control group. Treatment with high, medium or low doses of SHYCD reduced Bax and Cyt-C by different degrees, increased Bcl-2 protein levels, and increased Bax:Bcl ratio. Caspase -3 is the terminal shearing enzyme of cell apoptosis and plays an irreplaceable role in cell apoptosis. Caspase-3 enzyme generates two subunits, the 19,17 kD heterodimers. Cleaved caspase-3 is referred to as the death protease and is the last known apoptosis factor. Caspase-3 plays a central role in the process of apoptosis [48]. Apoptosis is the ultimate expression of genomic DNA fragmentation, resulting in an increased apoptosis index(AI). Figure 6 shows that the caspase-3 significantly increased and AI significantly decreased in the ACLF model group. Compared with the control group, groups treated with high, medium or low doses of SHYCD showed reduced caspase-3 and AI. Liver cell apoptosis is an extremely complex process, and pro-apoptotic protein p53 is a key regulator of the apoptotic process and is closely related to the regulation of apoptosis downstream proteins.

## CONCLUSIONS

SHYCD treatment improved the survival rate of rats, repaired liver function and blood coagulation, and repressed IL-1β, IL-6, and TNF-α expression. It’s possible mechanism of action is that SHYCD induces subcellular localization of APE1/Ref-1 to regulate the p53-apoptosis signaling pathway in the prevention and treatment of ACLF.

**Figure 7:**
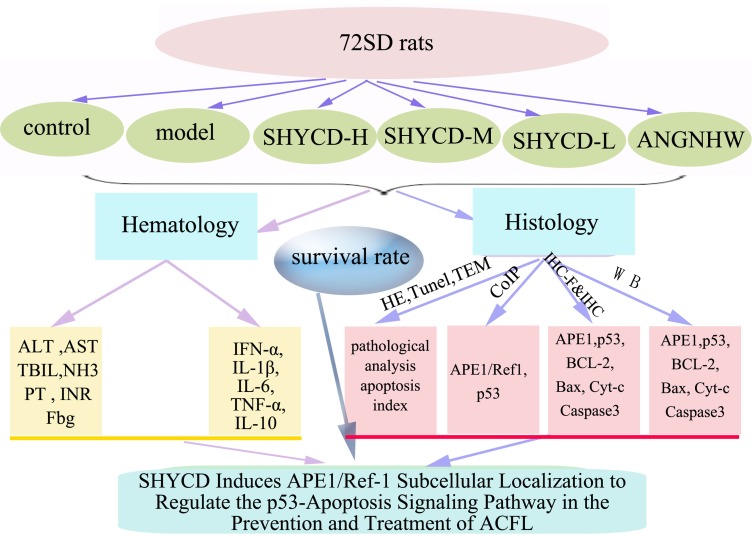
current working model

The current working model (Figure 7).

## MATERIALS AND METHODS

### SHYCD preparation

SHYCD was composed of five commonly used herbs: rhubarb, turmeric, *Aastragalus membranaceus*, Artemisia *capillaris*, and Radix Paeoniae *rubra*. The raw herbs were purchased from the Nanfang Hospital Affiliated to Southern Medical University (Guangzhou, China). The herbs were mixed in the ratio of 15:15:30:30:30 gram (dry weight). Aqueous extracts of SHYCD were obtained by stirring the herbs for 1 h with 8 volumes of distilled water (v/m) at 100 °C. The extracts were centrifuged at 1,500 g at room temperature. The supernatant was collected and condensed under reduced pressure at -80°C. SHYCD quality was confirmed by HPLC analysis (Supplementary Figure 1). SHYCD was suspended in 0.9% saline at a final concentration of 2 g/mL. The solution was stored in aliquots at -20°C.

### Animals and experimental procedure

All protocols were submitted to and validated by the Animal Care Ethics Committee of Southern Medical University(No. 2012–055). Pathogen-free SD rats, weighing 200–240g (certificate No. 4402102032),were purchased from the Experimental Animal Center of Southern Medical University. The animals were maintained under controlled conditions (22°C, 12h/12h dark/light cycle) in a conventional animal colony. Except for the control group(n = 10), 60 rats were injected with 40% CCL4 (2ml/kgbw,i.p.) peanut oil solution 2 times a week for 11 weeks. After 6 weeks, weekly B-ultrasound examination of liver size and amount of ascites was conducted. The other 5 groups, except for the control group, were injected with D-GalN (400 mg/kg, sigma i.p.) combined with LPS(10 ug/kg, sigma i.p.) for 4 weeks to establish the ACLF rat model [23]. We observed the rats’ activity and state. We recorded death circumstances within 48 hours. At the end of the study, there were 10 surviving rats in the control group, 6 in the model group, 8 in the SHYCD-H group, 8 in the SHYCD-M group, and 7 in the SHYCD-L group.

We selected 50 rats from the screened successful liver damage model. The rats were randomly divided into 5 groups: the model group, the SHYCD-H(30g/kg) group, the SHYCD-M(20g/kg) group, the SHYCD-L(10g/kg)group and the Angong Niuhuang Pill (0.5g/kg)group, each group of 10. once a day for 7 days of intragastric administration,The control group were administered intragastrically equivalent concentration of normal saline solution, The rats were killed by cervical dislocation after 7 days. Abdominal aortic blood was collected and the complete liver and spleen were removed and weighed. After gross examination to observe liver color and abdominal ascites, the livers were cut into pieces at about 1 cm intervals. The liver samples were then fixed in 10% buffered formalin (pH 7.4) or stored in 10 volumes of RNAlater Solution (Ambion, Life technologies, Carlsbad, CA, USA). The remaining samples were flash-frozen in liquid nitrogen and stored at -80°C for further Co-IP, PCR, and protein analysis.

### Blood examination

The serum levels of alanine aminotransferase (ALT), aspartate aminotransferase (AST), and total bilirubin (TBIL), and whole blood levels of prothrombin time (PT), blood coagulation time, international standard ratio (INR), and plasma fibrinogen(Fbg) were analyzed by the clinical laboratory of Nanfang Hospital Affiliated to Southern Medical University (Guangzhou, China). The serum levels of blood ammonia (NH3) were determined with an NH3 detection kit (Jiancheng, Nanjing, China).

### Histological examination and transmission electron microscopy (TEM)

A portion of liver was fixed in 10% formalin, embedded in paraffin, cut into 4 μm sections, mounted on slides, dewaxed, and rehydrated. The samples were stained with hematoxylin and eosin (HE) for histopathological examination. For TEM examination, a 2 mm portion was immediately fixed in 2.5% glutaraldehyde and 2% paraformaldehyde for 4 h, fixed with 1% osmium tetroxide for 2 h, dehydrated through a graded ethanol series, and then embedded in epoxy resin. Resin-embedded blocks were cut into 60 nm to 80 nm ultrathin sections with an ultramicrotome (PT-XL, RMC, USA). The ultrathin sections were placed on carbon-coated nickel grids and examined with an H-7500 transmission electron microscope (H-7500, Tokyo, Japan) operating at 80 kV.

### Cytokine measurement by multiplex immunoassay

Serum levels of IFN-α, IL-1β, IL-6, TNF-α, and IL-10 were detected with a mice cytokine multiplex kit (Millipore, Billerica, MA, USA). Cytokines quantification was performed with the Luminex system (Austin,TX, USA) [19].

### qPCR detection of APE1/Ref-1 mRNA expression

Total RNA was isolated from liver tissue with TRIzol® reagent (Takara Bio, China) dissolved in DEPC-treated water according to the manufacturer’s instructions. RNA (2 μg) wasreverse -transcribed to cDNA with oligo (dT) primers and PrimeScript RT Enzyme Mix reverse transcriptase (RT) in a final volume of 20 μl under the conditions recommended by the supplier (Takara Bio). For PCR amplification of APE/Ref1 and GAPDH, 1 μl of cDNA template and the following primers were used:APE/Ref1 forward 5’-TCA GAA AAC GTC AGC CAG TG-3’ reverse 5’-CGG GAG TTT GTT CTC TGA GC-3’; GAPDH forward 5’-ATT GTC AGC AAT GCA TCC TG-3’, reverse5’-ATG GAC TGT GGT CAT GAG CC-3’. The reaction cycle conditions were: denaturation at 90°C for 30 s, annealing at 60°C for 30 s, and extension at 72°C for 30 s. APE/Ref1 expression levels were normalized to that of GAPDH, which was used as a specific endogenous control. Real-time data collection and quantitative analysis were performed with Strategen Mx3000p software.

### Western blot

Total and nuclear protein were prepared individually from livers with kits for tissue and nuclear protein extraction (Pierce Biotechnology, Rockford, IL,USA), according to the manufacturer’s instructions. Protein concentration was determined by BCA (Pierce Biotechnology, Rockford, IL,USA). SDS-PAGE analysis was conducted with 50 μg liver protein on a 10% gel. Bands were electro -transferred to PVDF membranes (Merck Millipore, Germany) and blocked in 5% non-fat milk in Tris-buffered saline solution (TBST, 100 mM NaCl, 50 mM Tris,0.1% Tween-20, pH 7.5). Membranes were incubated overnight with primary antibodies. APE1/Ref-1 (Abcam Biotechnology, USA, dilution 1:1000), p53 (Cell Signaling Technology, CST dilution 1:500), caspase-3, Cyt-c, Bcl-2, and Bax (Cell Signaling Technology,CST dilution1:1000.) at 4°C. Membranes were then incubated with horseradish peroxidase (HRP) conjugated secondary antibodies. Protein bands were visualized with enhanced chemiluminescence reagents (ECL, Merck Millipore, Germany). Images were captured with a CCD system (Imagestation 2000 MM, Kodak, Rochester, NY, USA). Quantitative analysis of signals was performed with Molecular Imaging Software Version 4.0, provided by Kodak 2000 MM System. Optical density was normalized to β-actin.

### Immunofluorescent *in situ* detection of APE1/Ref-1 and P53 protein expression

Liver sections were dewaxed in xylene, dehydrated in a gradient alcohol series, then washed three times with PBS for five minutes each time. The sections were microwaved in citrate and cooled at room temperature. Sections were incubated in 1% Triton-100 for 5 min, incubated in 3% H2O2 endogenous blocking for 10 min at room temperature, then incubated in 5% normal goat serum and a solution of primary antibodies specific to APE1/Ref-1(Abcam, USA, dilution 1:50) and p53 (Cell Signaling Technology, CST dilution 1:200) for 30 min. The sections were placed in a humidified chamber and incubated overnight at 4 °C. The sections were washed with PBS washed three times for 5 min each time. The sections were incubated with the second immunofluorescence antibody (dark) for 1 h at 37 °C. The sections were washed with PBS three times for washed 5 min each time. DAPI -stained nuclei were incubated at room temperature for 10min. Stained sections were washed with PBS three times for 5 min each time. We added 5–10 µl fluorescence decay-resistant sealing tablets. The sections were observed with a confocal microscope.

### Immunoprecipitation(CoIP)

CoIP assay was conducted according to the manufacturer’s instructions (Roche, Mannhein, Germany). Briefly, lysis buffer was added to total protein extracted by centrifugation. The total protein extract was incubated with anti-APE1 antibody at 4°C overnight. Agarose beads were added at a ratio of 1 mg of extract per 30 ml of agarose at 4°C for 3 h. The beads were then pelleted at 2,500 × g for 3 min and washed with lysis buffer 5 times. The beads were eluted with 5 vol of 0.5 mg/ml peptide for 4 h or boiled at 100°C for 3 min and subjected to SDS gel electrophoresis. After silver staining, bands appearing in the experimental group were excised and sent for mass spectrometry analysis.

### Immunohistochemistry

Immunohistochemical staining for caspase-3,Cyt-c, Bcl-2, and Bax was performed by routine immunohistochemistry streptavidin peroxidase method. This method utilized a rabbit polyclonal IgG antibody against caspase-3, Cyt-c, Bcl-2, and Bax (Cell Signaling Technology,CST; dilution 1:200). Nuclear counterstaining was performed with hematoxylin. Five randomly selected fields from each section were examined at a magnification of 200x and analyzed with Image-Pro Plus 6.0. The positive content (PC) was calculated as (PC) = mean optical density × positive area[24].

### Apoptosis assay

We purchased a terminal deoxynucleotidyl transferase dUTP nick end labeling (TUNEL) apoptosis assay kit (Roche, Switzerland). We performed all the procedures based on the manufacturer’s instructions. Cells were defined as apoptotic if the entire nuclear area of the cell was positively labeled. The apoptotic index (AI) was calculated as (AI) = number of apoptotic cells/total number of nucleated cells [13].

### Statistical analysis

Each experiment was repeated at least three times. Data were represented as means ± SD. Data were analyzed with SPSS statistical package (version 13.0, Armonk, NY, USA). Mean values were compared by one-way ANOVA. Multiple comparisons were performed. Data were analyzed by a homogeneity test for variance. If the variances were homogeneous, mean values were compared through ANOVA. The differences between two groups were analyzed based on the Least Significant Difference test. If the variances were not homogeneous, the mean values were compared by Welch’s test. The differences between two groups were analyzed by Games-Howell. Statistical significance was set at *P* < 0.05.

## SUPPLEMENTARY MATERIALS FIGURE



## Abbreviations

ACLF: acute on chronic liver failure
SHYCD: Sanhuangyinchi decoction
CCL4: Carbon tetrachloride
D-GalN: D-galactosamine
LPS: lipopolysaccharide
APE1/Ref-1: Apurinic/apyrimidinic endonuclease/redox effector factor-1
HPLC: High Performance Liquid Chromatography
ALT: Alanine aminotransferase
AST: Aspartate transaminase
TBIL: total bilirubin
NH3: Ammonia
IL-6: interleukelin-6
PT: prothrombin time
ROS: reactive oxygen species
TNF-α: tumor necrosis factor-alpha
Bcl-2: B-cell lymphoma 2
Cyt-c: Cytochrome-c
