# Supplementary material for: SHYCD induces APE1/Ref-1 subcellular localization to regulate the p53-apoptosis signaling pathway in the prevention and treatment of acute on chronic liver failure
Source: Oncotarget. 2017 Aug 4;8(49):84782–97. doi: 10.18632/oncotarget.19891 (PMC5689573; doi:10.18632/oncotarget.19891)
Supplement: Supplementary file 1 [file oncotarget-08-84782-s001.pdf]

SHYCD induces APE1/Ref-1 subcellular localization to regulate the p53-apoptosis signaling pathway in the prevention and treatment of acute on chronic liver failure

SUPPLEMENTARY MATERIALS

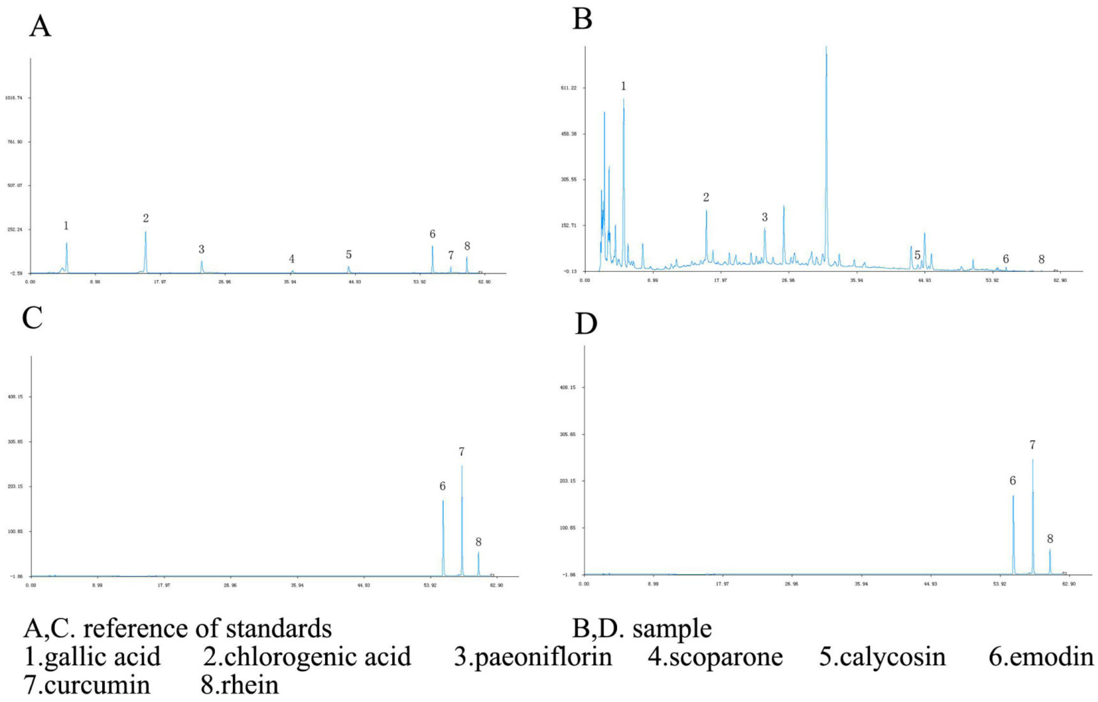

Supplementary Figure 1: chromatograms of reference of standards, sample at 280, 430nm, respectively. A, **C**:reference of standards **B**, **D**:sample. 1.gallic acid ; 2.chlorogenic acid; 3.paeoniflorin 4.scoparone; 5.calycosin; 6.emodin; 7.curcumin 8.rhein
